# Bibliometric analysis of trends and hotspots in thyroid hormone research in patients with bipolar disorder (1998–2022): Bibliometric review

**DOI:** 10.1097/MD.0000000000049934

**Published:** 2026-07-31

**Authors:** Xiuhua Song, Lei Yi, Shenghai Wang, Ligang Wang

**Affiliations:** a Department of Psychiatry, Mental Health Center of Qingdao City, Qingdao, Shandong Province, China.

**Keywords:** bibliometrics, bipolar disorder, CiteSpace, thyroid hormones, VOSviewer

## Abstract

**Background:**

Bipolar disorder (BD) is a chronic psychiatric illness. Thyroid hormones play a crucial role in maintaining normal brain function and have a significant impact on mood regulation and neuroendocrine activity. Increasing evidence suggests that thyroid dysfunction is closely related to the onset, progression, and severity of BD. A systematic bibliometric analysis of this field is important for understanding its research trajectory and identifying emerging hotspots.

**Methods:**

Literature on BD and thyroid hormones published between 1998 and 2022 was retrieved from the Web of Science Core Collection. Data were analyzed and visualized using VOSviewer and CiteSpace software to explore publication trends, major contributing countries and institutions, authors, journals, and keyword characteristics.

**Results:**

A total of 452 relevant publications were identified, covering 229 journals, 2318 authors, 1443 institutions, and 188 countries. The United States, China, and Germany were the most active countries in this field. Major contributing institutions included the University of California, Los Angeles, the University of Toronto, and the Technical University of Dresden. Leading scholars were Bauer M, Whybrow PC, and Rybakowski JK. The Journal of Affective Disorders published the most relevant papers. Keyword analysis revealed that research hotspots mainly focused on BD, thyroid function, and depression, particularly around themes such as “risk,” “affective illness,” and “bipolar affective disorder.”

**Conclusion:**

The relationship between thyroid hormones and BD has become an important research focus in recent years, providing new directions for future studies in this field. This study systematically reveals the research landscape and developmental trends from 1998 to 2022, offering valuable references and guidance for future research and clinical practice.

## 1. Introduction

According to the International Classification of Diseases, 10th Edition diagnostic criteria, bipolar disorder (BD) is classified into manic episodes, depressive episodes, mixed states, and other subtypes. With growing awareness of the disease, scholars’ interest in researching BD has increased annually. The exact etiology of BD remains incompletely understood, but it is considered to be the result of a combination of genetic, environmental, and neurobiological factors. Thyroid hormones play a critical role in maintaining normal brain function and can modulate mood and behavior. Accumulating evidence suggests that thyroid hormone dysregulation may be associated with the development, progression, and severity of BD.

The association between affective psychosis and thyroid disorders was first noted in 1888.^[1]^ By the 1980s, scholars had identified a close link between thyroid axis abnormalities and affective disorders and found that subclinical hypothyroidism was the most common condition in patients with rapid-cycling BD.^[2,3]^ In 1998, a study reported that thyroid axis abnormalities were more frequent in patients with bipolar mixed states than in those with bipolar mania.^[4]^ Since then, the investigation in this area has expanded steadily. The present study uses a bibliometric approach to review and summarize literature on thyroid hormones in BD, aiming to delineate the current research landscape and hotspots and provide references for future studies in this field.

## 2. Methods

### 2.1. Search strategy and data collection

**Figure 1.**
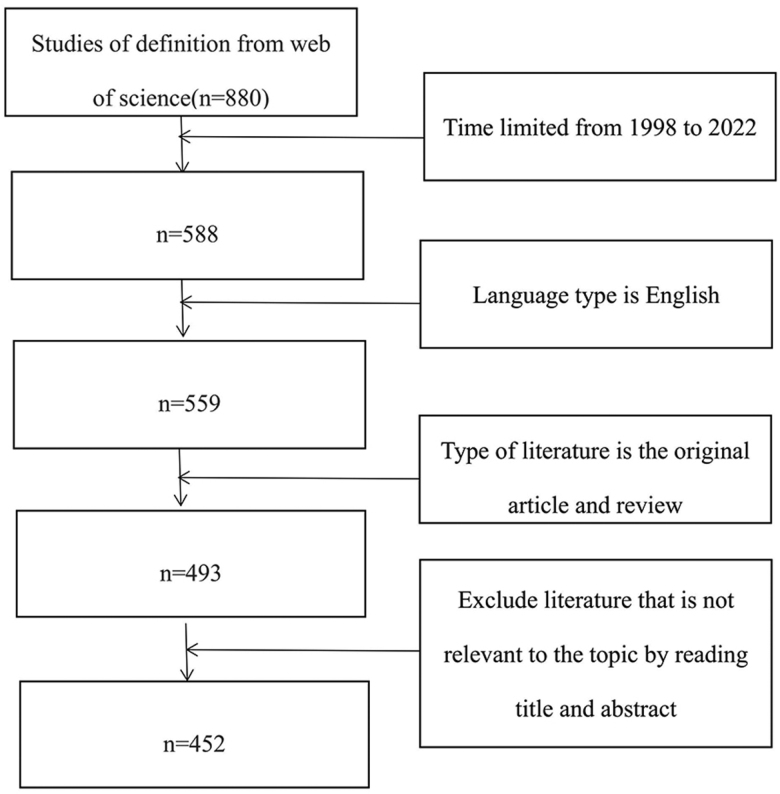
Flowchart depicting the article selection process.

All data used in this study were obtained from the Web of Science database. The Web of Science Core Collection (WoSCC) served as the data search platform, and the search formula was “TS = (bipolar disorder OR bipolar depression OR bipolar mania OR bipolar disorder rapid cycling OR bipolar mixed states) AND (thyroid hormone OR thyroid function OR thyroxine OR thyroid dysfunction).” The time span was from January 1, 1998, to December 31, 2022. Inclusion criteria: literature focusing on the association between BD and thyroid hormones; literature types: original articles and reviews; language: English. Exclusion criteria: duplicate literature or studies unrelated to the research topic; literature from conference proceedings, case reports, books, letters, etc. In this study, all data used were sourced from a public database, rendering ethics committee consent or institutional review board approval unnecessary. The detailed screening process was illustrated in Figure 1, and a total of 452 eligible documents were finally included.

### 2.2. Data analysis

VOSviewer (a powerful visualization tool developed by Leiden University) is utilized to analyze and visualize the co-occurrence network of keywords, intercountry, and inter-institution collaboration axes.^[5]^ Node sizes in VOSviewer are indicative of their frequency of appearance in both titles and abstracts.^[6]^ It is worth mentioning that VOSviewer is selected because of its capability to produce more structured mappings than other widely used bibliometric software.^[5]^ In this study, VOSviewer was used to conduct a bibliometric analysis of the retrieved literature. Specifically, the software was applied to construct visualized bibliometric networks, including journals, authors, and individual publications. These networks were generated based on citation, bibliographic coupling, co-citation, and co-authorship relationships. In addition, based on the bibliographic data, VOSviewer was utilized to create the keywords’ co-occurrence, also known as the co-words overlay visualization. We employed the text-mining function of VOSviewer to extract significant terms from the dataset, which allowed us to create and visualize co-occurrence networks. This process enabled the identification of thematic clusters and the mapping of research hotspots within the field.

VOSviewer employs a distance-based visualization method that is highly effective in presenting knowledge graphs. In contrast, CiteSpace (version 6.1.R.6), a widely used tool for knowledge mapping, uses clustering algorithms to identify emerging terms and assist researchers in identifying research trends and new directions. In this study, CiteSpace was employed to conduct bibliometric and visualization analyses. It automatically identifies research frontiers based on citation node literature and co-citation clusters, providing a basis for knowledge domain analysis.^[7]^ The CiteSpace Operation Steps are as follows: create 4 folders: input, output, data, and project; export literature from the Web of Science database in download.txt format and save it to the input folder; use CiteSpace to process the data, which is then saved to the output folder; copy the processed data to the data folder. Create a new project in CiteSpace, set the time slice to 1 year (covering 1998–2022), and select node types (e.g., author, institution, country/region, keyword, cited literature, cited author, cited journal); generate visual graphs of author collaboration networks and institutional collaboration networks, and perform cluster analysis on high-frequency keywords to identify research hotspots. In the CiteSpace analysis, each cluster was labeled by noun phrases from the titles of the citing articles in the cluster. The label of each cluster was extracted with a log-likelihood ratio test, which summarizes the impact of the cluster on more recent research. In addition, the cluster relationship could be interpreted through the modularity *Q* value. The mean silhouette *S* value informed the quality of a clustering configuration. Herein, modularity *Q* was 0.8476, and mean silhouette *S* was 0.9536. The tool used to create network visualization reveals cluster overlap, indicating potential conceptual similarities across different research streams. This overlapping may arise due to similar methodological approaches, shared foundational literature, or overlapping research topics examined from different disciplinary perspectives. However, the size and structure of these clusters indicate varying degrees of thematic similarity among the grouped publications, suggesting the concentration of research activity in specific thematic areas.

## 3. Results

Research on BD and thyroxine has a total of 452 publications from 1998 to 2022, involving 229 journals and a total of 2318 authors, including 1443 organizations in 188 countries.

### 3.1. Time distribution

**Figure 2.**
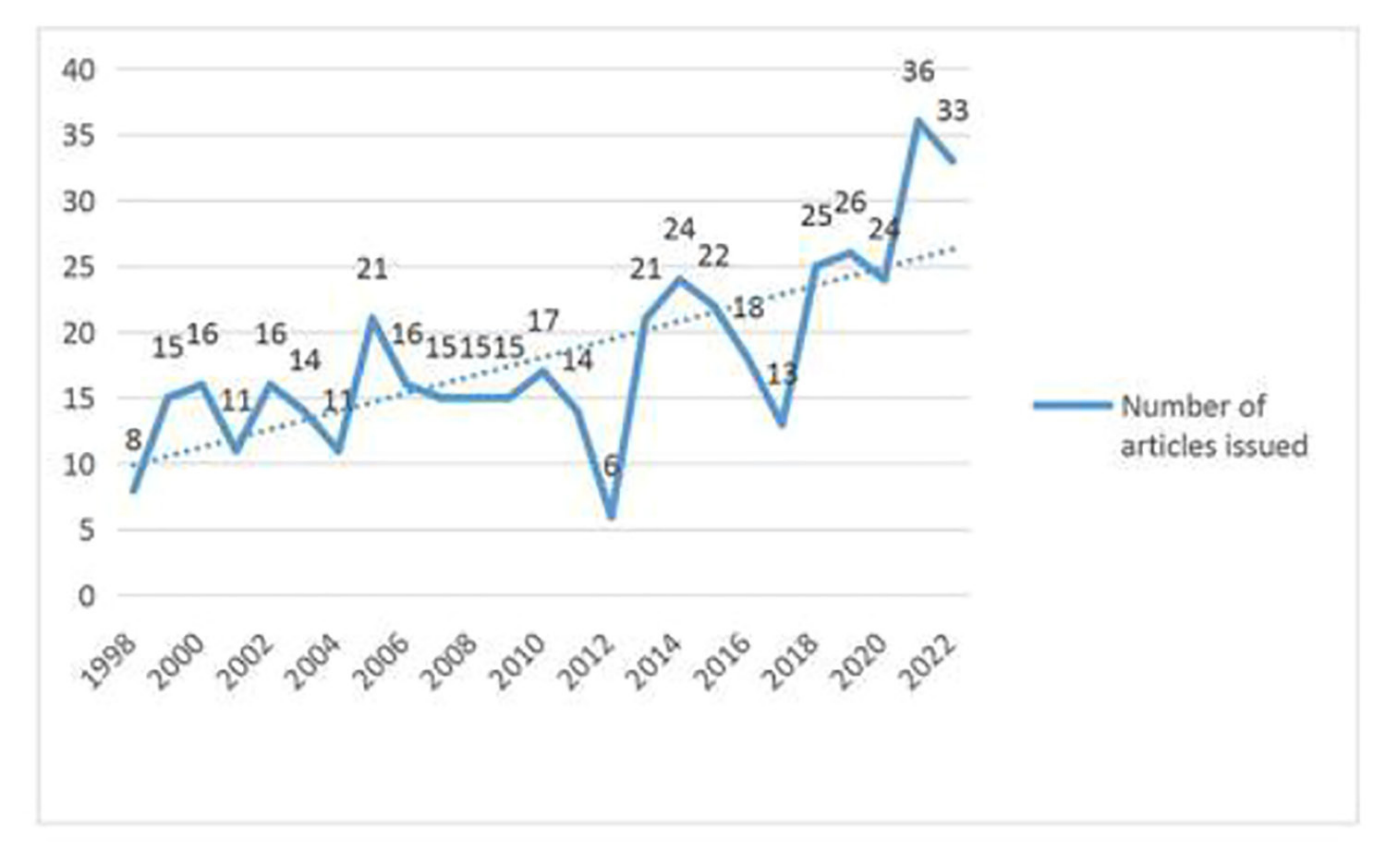
Annual volume of publications.

After converting data using CiteSpace and importing it into Excel, a line graph of annual publication volume was generated (Fig. 2). The number of studies on BD and thyroid hormones showed an overall upward trend from 1998 to 2022: the highest number of publications (36 articles) was recorded in 2021, and the lowest (6 articles) in 2012.

### 3.2. Country distribution

**Figure 3.**
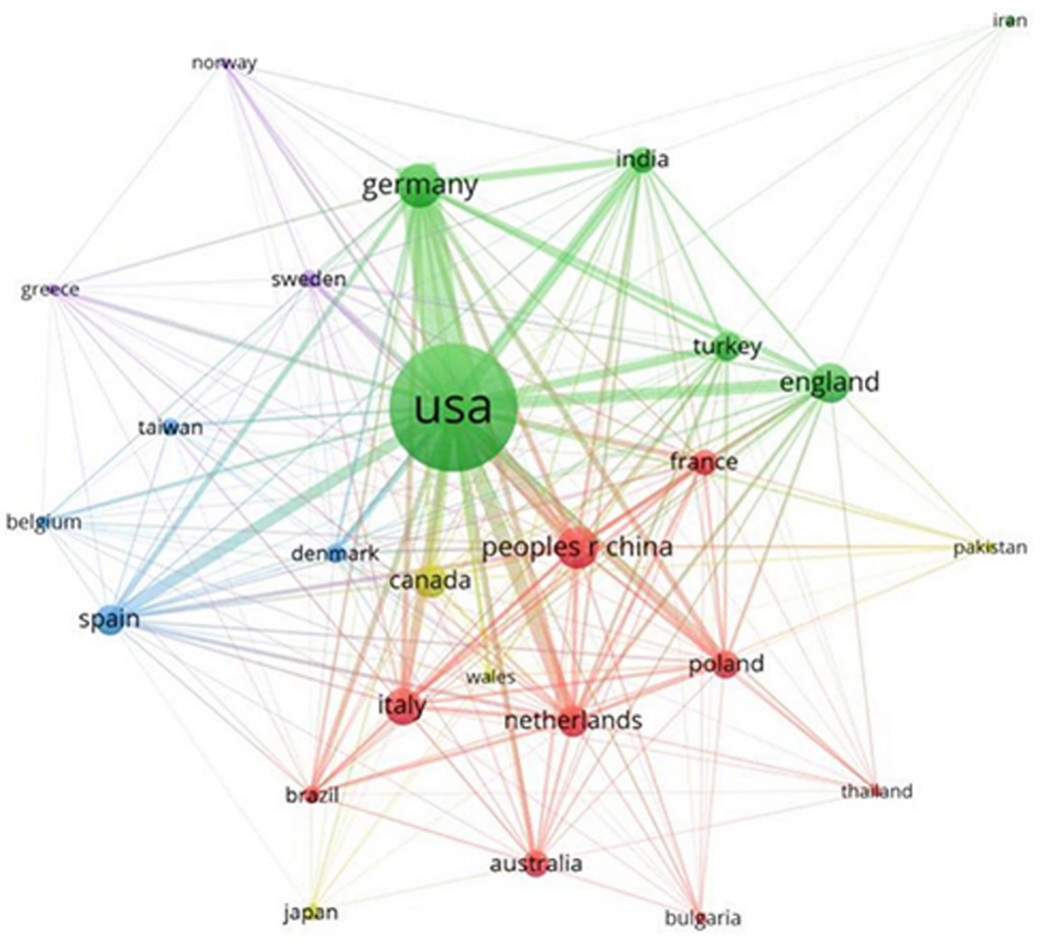
Countries on the list for studying bipolar disorder.

VOSviewer was used to analyze highly cited countries, with the minimum number of publications set to 5 and the default minimum citation count set to 0. Among the 188 countries with relevant publications, 26 met the criteria (Fig. 3). The study revealed that these countries were the primary focus of researchers when examining the topic. Researchers often prefer to cite these countries when conducting further research.

As shown in Table 1, the United States was the most active country, with 169 publications (accounting for 37.38% of the total), followed by China and Germany. Other countries in the top 10 in terms of the number of publications are the UK, Israel, Canada, the Netherlands, Spain, Turkey, and Australia (Table 1). In the labeled view of VOSviewer, darker colors indicate an older average publication year: the United States, Germany, and the United Kingdom were among the first countries to initiate research in this field. Although France did not rank in the top 10 in terms of the number of publications and citations, its literature should still be considered when reviewing the field. Canada, the Netherlands, and Spain also began researching this topic relatively early.

**Table 1:** The top 10 productive countries related to bipolar disorder and thyroid hormone.

Ranking	Country	Article counts	Percentage	Citations	Total link strength
1	USA	169	37.38%	6677	1195
2	China	46	10.18%	545	367
3	Germany	38	8.41%	1236	634
4	England	33	7.30%	1671	239
5	Italy	30	6.64%	1021	194
6	Canda	26	5.75%	1042	166
7	Netherlands	24	5.31%	982	319
8	Spain	22	4.87%	436	201
9	Turley	21	4.65%	315	163
10	Austrilia	19	4.20%	373	97

Total link strength is applied to evaluate the overall connectivity among nodes, helping to reveal knowledge structures, collaboration patterns, and research hotspots. Higher total link strength values indicate more active and extensive connections, often pointing to core nodes in the network. Among the top 10 posting countries, the US has 6677 citations, which is much higher than those of other countries, while its total link strength is 1195. Although China had a relatively high publication volume, it only had 545 citations and a total link strength of 367. Notably, France – despite not being in the top 10 by publication count – had 1067 citations (higher than China), indicating higher quality in its literature.

### 3.3. Institutional distribution

**Figure 4.**
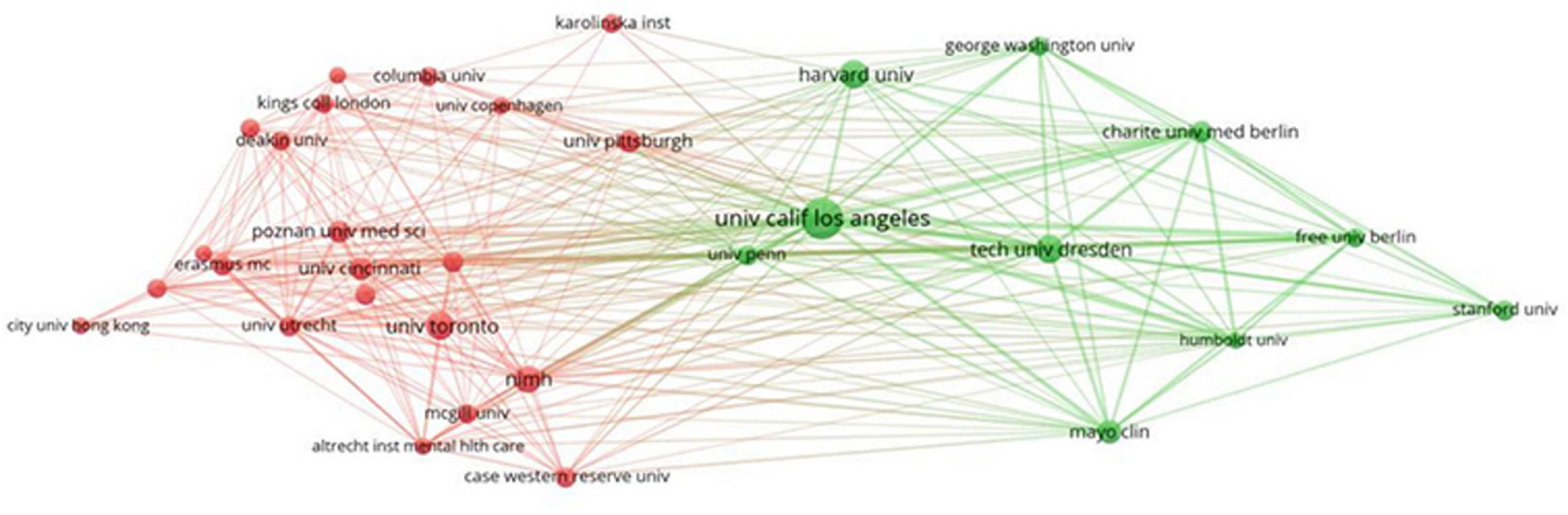
Top10 organization distribution.

Analysis of publishing institutions in the field of thyroid hormone research in BD showed that, except for the National Institute of Mental Health (NIMH) and Mayo Clinic, the top 10 institutions by publication count were all universities (Table 2). The University of California, Los Angeles (United States) ranked first with 29 publications, followed by the University of Toronto (Canada), Technical University of Dresden (Germany), and Harvard University (United States). The network of cooperation is not independent, and there is more cross-collaboration. Major collaborative networks included those centered on the University of California, Los Angeles, the University of Toronto, and the Technical University of Dresden (Fig. 4).

**Table 2:** Top 10 institutions in terms of number of articles published.

Ranking	Institution	Country	Article counts	Citations	Total link strength
1	University of California Los Angeles	United States	29	1456	501
2	University of Toronto	Canada	14	714	45
3	Technical University of Dresden	Germany	13	293	302
4	Harvard University	United States	13	560	64
5	NIMH	United States	12	1116	104
6	Mayo Clin	United States	10	169	137
7	Univ Cincinnati	United States	9	620	69
8	Poznan Univ Med Sci	Poland	9	198	55
9	Univ Pittsburgh	United States	9	575	43
10	Charite Univ Med Berlin	Germany	8	251	203

NIMH = National Institute of Mental Health.

### 3.4. Journal distribution

**Figure 5.**
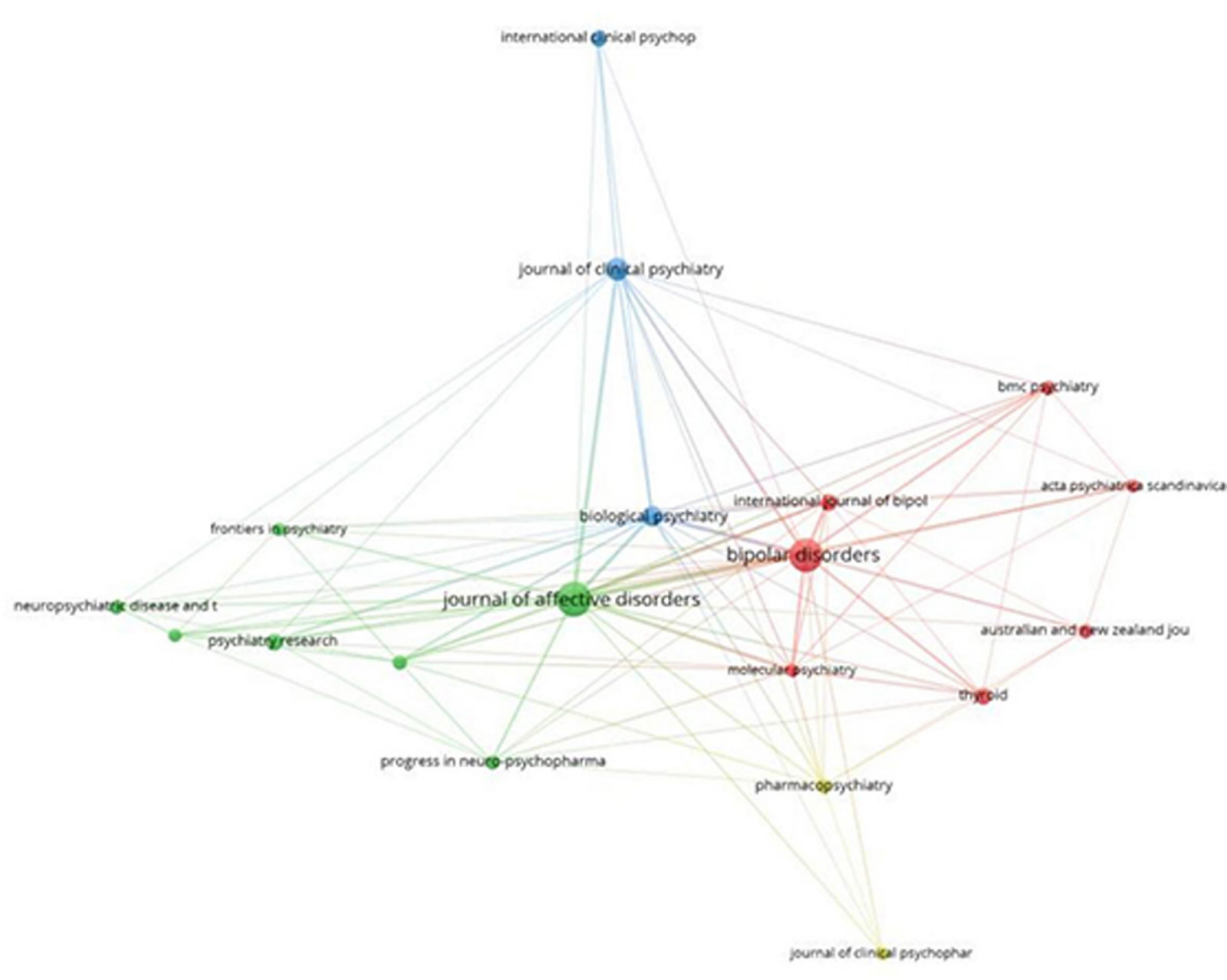
Distribution of journals.

The 3 journals with the highest number of publications were the Journal of Affective Disorders (31 articles), Bipolar Disorders (29 articles), and the Journal of Clinical Psychiatry (13 articles). Detailed information on the top 5 journals by publication count is shown in Table 3 and Figure 5.

**Table 3:** Top 5 journals in terms of number of articles published.

Ranking	Periodicals	Article counts	Citations	Total link strength
1	Journal of Affective Disorders	31	707	125
2	Bipolar Disorders	29	1162	118
3	Journal of Clinical Psychiatry	13	702	49
4	Biological Psychiatry	11	948	78
5	International Journal of Bipolar Disorders	7	270	42

### 3.5. Author distribution and author collaboration network

**Figure 6.**
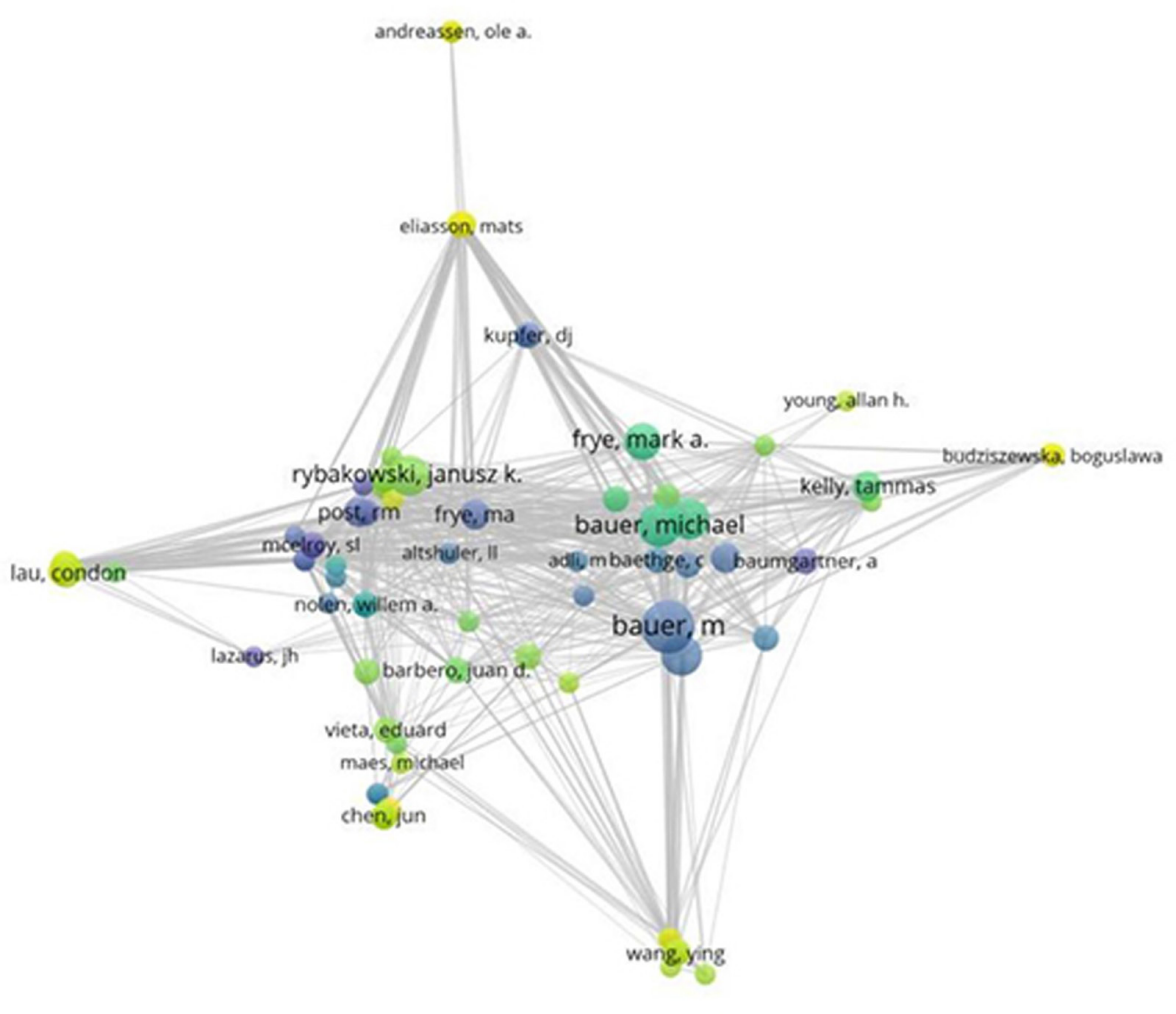
Author and author partnership.

The number of publications and citations reflects the influence of key scholars in the field. As shown in Table 4 and Figure 6, Bauer M (University of California, Los Angeles), Whybrow PC (University of California, Los Angeles), and Frye Mark A (University of California, Los Angeles) ranked in the top 3 by publication count, with 21, 16, and 12 articles, respectively. The top 10 cited authors were mainly from the United States, Germany, and other countries.

**Table 4:** Top 10 most prolific writers.

Ranking	Author	Article counts	Citations	Total link strength
1	Bauer M	21	928	834
2	Whybrow PC	16	560	663
3	Frye Mark A	12	559	339
4	Rybakowski JK	8	117	124
5	Berghofer AD	5	178	193
6	Post RM	5	395	135
7	Kelly Tammas	5	62	136
8	Lau Condon	5	25	8
9	Baethe C	4	114	15
10	Bschor T	4	146	15

### 3.6. High frequency keyword clustering and time zone map

**Figure 7.**
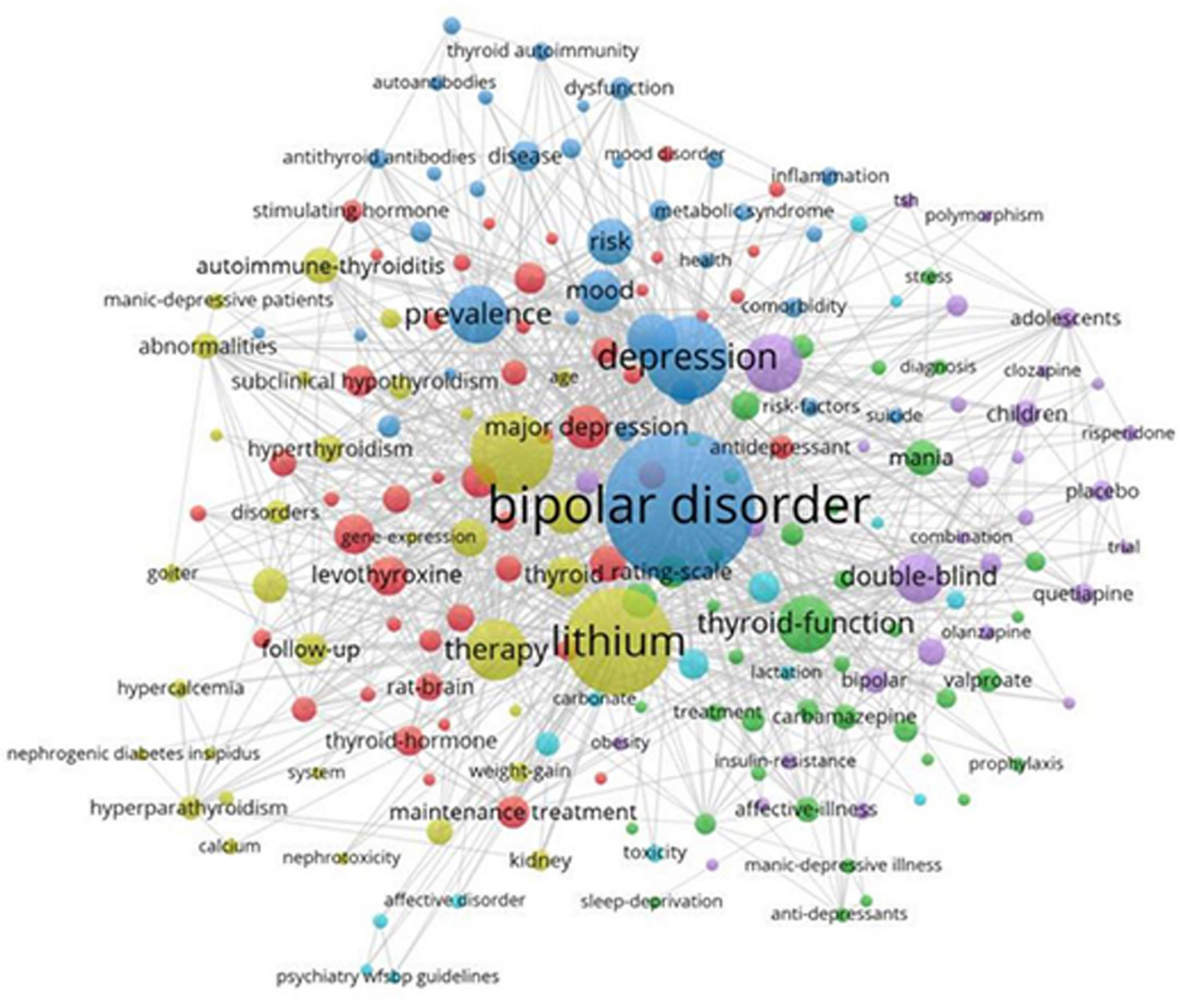
Keyword co-occurrence network relationship diagram.

The frequency of keyword occurrence reflects the researcher’s attention to a certain content, and high-frequency keywords reflect hot topics in the research field. The keywords of thyroid hormone research literature in BD patients were counted, and the top 10 high-frequency keywords are shown in Table 5. The keyword co-occurrence network relationship diagram using VOSviewer is shown in Figure 7.

**Table 5:** Top 10 keyword rankings.

Ranking	Citation	Centrality	Year	Keywords
1	220	0.31	1998	Bipolar disorder
2	55	0.16	1999	Thyroid function
3	47	0.10	1998	Depression
4	45	0.09	1999	Therapy
5	40	0.20	2000	Thyroid hormone
6	40	0.09	1998	Hypothyroidism
7	37	0.14	1999	Association
8	37	0.06	2002	Prevalence
9	33	0.10	1998	Disorder
10	32	0.10	1999	Double blind

**Figure 8.**
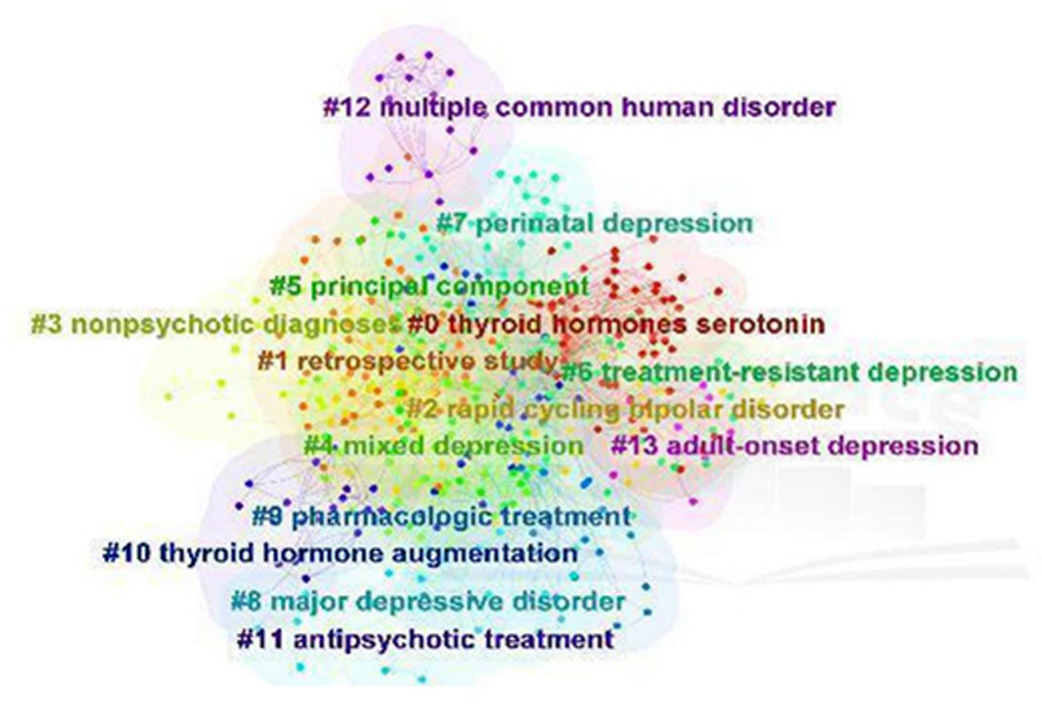
Keyword clustering.

**Figure 9.**
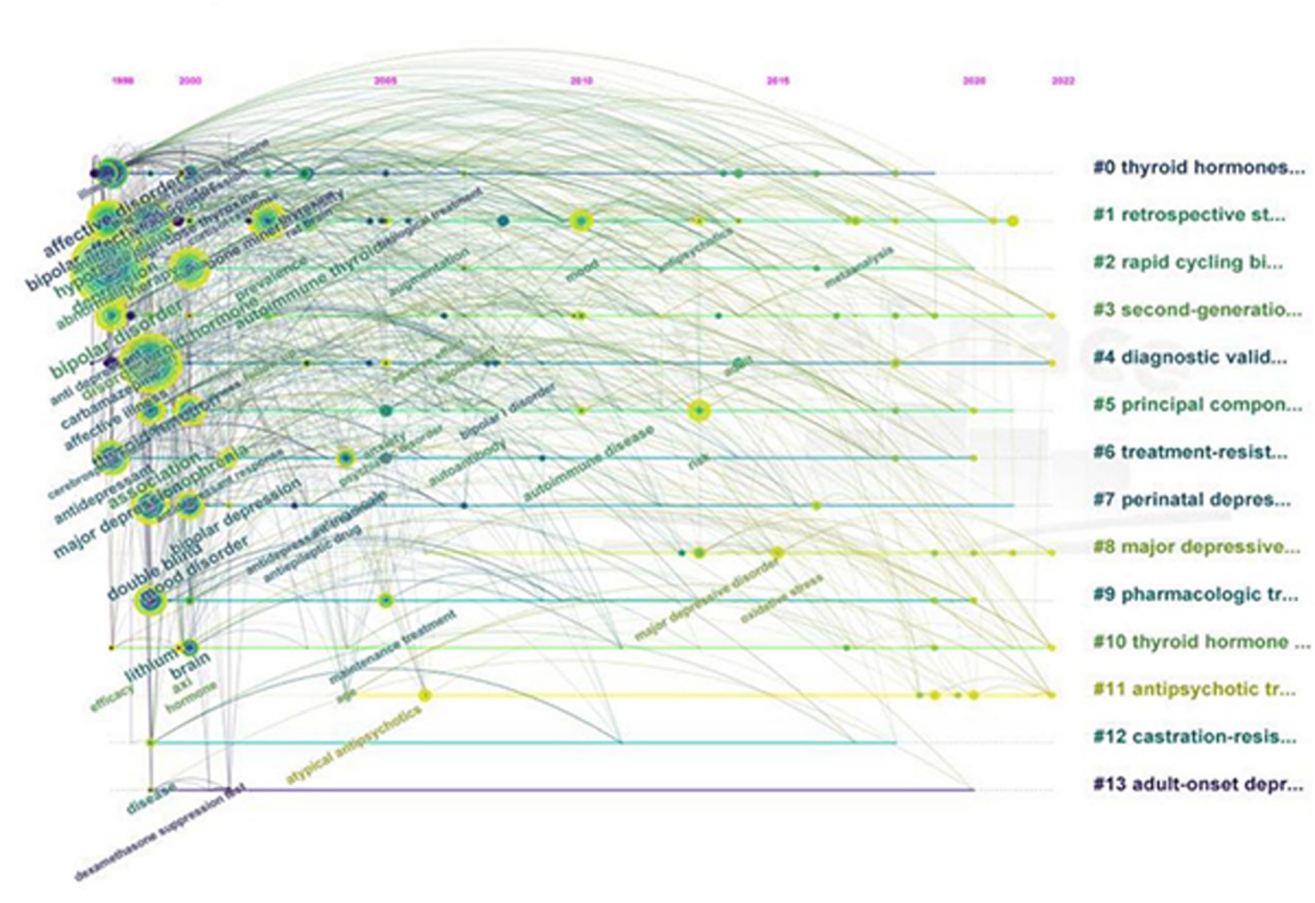
Keyword time zone map.

CiteSpace was used to cluster keywords and analyze research hotspots (Figures 8,9, Table 6). The 14 main clusters and their core keywords are as follows: thyroid hormones and serotonin-related studies: bipolar affective disorder, affective disorder, and bone mineral density, among others. Retrospective study-related studies: depression, therapy, hypothyroidism, etc. Rapid cycling BD: BD, thyroid hormone, abnormality, etc. Neuroendocrine network-related research: disorder, follow-up, children, etc. Rapid antidepressant response-related research: thyroid function, affective illness, carbamazepine, etc. Principal component-related studies: association, schizophrenia, risk, etc. Treatment-resistant depression-related research: major depression, bipolar depression, rating scale, etc. Perinatal depression-related studies: double-blind, mood disorder, subclinical hypothyroidism, etc. Major depressive disorder-related studies: oxidative stress, major depressive disorder, brain development, etc. Pharmacologic treatment-related research: lithium, brain, maintenance treatment, etc. Thyroid hormone augmentation-related research: hormone, axis, efficacy, etc. Antipsychotic treatment-related research: stimulating hormone, atypical antipsychotics, insulin resistance, etc. Multiple common human disorder-related research: disease, Child Behavior Checklist (CBCL) dysregulation profile, colorectal cancer, etc. Adult-onset depression-related research: dexamethasone suppression test, growth hormone secretion, corticotropin-releasing factor. The time zone map further reveals the evolving trend of research topics over time. It starts with early studies focused on pathophysiological mechanisms and the classification of mood disorders, gradually transitioning toward pharmacological mechanisms, hormonal treatments, and the optimization of clinical intervention strategies. This evolution reflects a deepening and multidimensional understanding of the relationship between BD and thyroid hormones.

**Table 6:** Summary of the largest 14 clusters.

ClusterID	Size	Silhouette	Keywords	Label (LLR)	Average year
0	62	0.712	32 Bipolar affective disorder 25 affective disorder 13 bone mineral density	Thyroid hormones serotonin	2004
1	62	0.755	47 Depression 45 therapy 40 hypothyroidism	Retrospective study	2008
2	52	0.77	220 Bipolar disorder 40 thyroid hormone 15 abnormality	Rapid cycling bipolar disorder	2010
3	42	0.791	33 Disorder 11 follow up 9 children	Neuroendocrine network	2012
4	42	0.806	55 Thyroid function 14 affective illness 12 carbamazepine	Rapid antidepressant response	2005
5	39	0.833	37 Association 27 schizophrenia 26 risk	Principal componenti	2009
6	36	0.875	25 Major depression 23 bipolar depression 14 rating scale	Treatment-resistant depression	2007
7	32	0.81	32 Double blind 23 mood disorder 7 subclinical hypothyroidism	Perinatal depression	2006
8	27	0.85	11 Oxidative stress 11 major depressive disorder 3 brain development	Major depressive disorder	2015
9	26	0.853	26 Lithium 14 brain 10 maintenance treatment	Pharmacologic treatment	2009
10	26	0.788	12 Hormone 8 axi 6 efficacy	Thyroid hormoneaugmentation	2012
11	20	0.876	8 Stimulating hormone 8 atypical antipsychotics 6 insulin resistance	Antipsychotic treatment	2018
12	14	0.877	10 Disease 1 CBCL dysregulation profile 1 colorectal cancer	Multiple common human disorder	2011
13	10	0.924	5 Dexamethasone suppression test 2 growth hormone secretion 2 corticotropin releasing factor	Adult-onset depression	2004

CBCL = Child Behavior Checklist; LLR = log-likelihood ratio.

### 3.7. Keyword burst analysis

Keyword burst analysis identifies terms that have gained sudden attention in the field. The top 10 burst keywords are shown below (burst intensity in parentheses, followed by cluster and year):

“Risk” (6.42, Cluster #5, 2013)

“Affective illness” (6.21, Cluster #4, 1998)

“Rat brain” (5.03, Cluster #0, 2003)

“Bipolar affective disorder” (4.98, Cluster #0, 1998)

“Prevalence” (4.07, 2002)

“Carbamazepine” (4.05, Cluster #4, 1998)

“Lithium” (4.05, Cluster #4, 1999)

“Follow up” (3.30, Cluster #3, 2002)

“High dose thyroxine” (3.25, Cluster #0, 2000)

“Disorder” (3.18, Cluster #4, 2000)

Among these, “risk” exhibited the strongest burst intensity (6.42), indicating that risk-related factors – such as genetic predisposition, treatment-induced thyroid dysfunction, and comorbidity risks – became a major focus of research beginning in 2013. The early bursts of “affective illness” and “bipolar affective disorder” around 1998 mark the initial phase of systematic exploration into the neurobiological basis and clinical management of affective disorders. The keyword “rat brain” (2003) reflects the transition toward preclinical and mechanistic studies, emphasizing animal models used to investigate the neuroendocrine and neurochemical mechanisms underlying BD.

The appearance of “carbamazepine” and “lithium” as burst keywords during the late 1990s indicates the historical focus on mood stabilizers, which laid the foundation for the pharmacological management of BD. Meanwhile, “high dose thyroxine” emerging around 2000 suggests a growing research interest in thyroid hormone augmentation therapy, particularly the use of supraphysiologic levothyroxine (L-T4) in treatment-resistant mood disorders. The term “follow up” reflects an increasing emphasis on longitudinal studies and treatment outcomes, highlighting the need for sustained monitoring of thyroid and mood status in bipolar patients.

Overall, the burst keyword analysis demonstrates a temporal shift in research focus – from traditional pharmacotherapy and affective mechanisms toward integrative neuroendocrine regulation and personalized treatment approaches. This evolution underscores the interdisciplinary nature of BD research, linking psychiatry, endocrinology, and neurobiology.

## 4. Discussion

Limited attention has been given to how research clusters evolve over time, and to what extent they address critical clinical challenges such as the association between thyroid hormone, BD, and BD treatment. To address this gap, this study employs bibliometric cluster analysis to map global research trends, identify thematic clusters, and characterize the interplay between basic research and clinical implementation. Specifically, our study aims to address the informational and thematic gaps in scientific production on clinical communication, as indexed in the WoSCC, from 1998 to 2022. This study is the first bibliometric analysis of BD and thyroid hormones in nearly 20 years, revealing an upward trend in annual publication volume. It is noted that the highest number of publications (36 articles) was recorded in 2021. For example, Shunkai Lai et al found that how thyroid hormones interact with the neurometabolic aspects of the prefrontal cortex–thalamus circuitry to result in cognitive impairment in patients with bipolar type 2 depression needed to be investigated.^[8]^ In the study conducted by Ingrid Lieber et al,^[9]^ it currently remains unclear how thyroid hormone replacement therapy is used in patients with bipolar depression. Equally, they do not know whether a comparable trend of thyroid hormone replacement therapy prescription has occurred. In another study, Shengnan Zhao et al assessed the thyroid function of drug-naive BD across different mood states, with the expectation of providing support for treatment options.^[10]^ Thus it can be seen that the uncertainty between thyroid hormones, cognitive impairment, bipolar depression, and thyroid hormone replacement therapy may be underlying factors driving these fluctuations in the upward trend in annual publication volume, especially in 2021. In addition, the increase in the number of published articles in 2021 may be related to the increase in research funds, such as the Research and Development Fund of Norrbotten Region, Northern County Councils Regional Federation, and West China Psychiatric Association.

The United States was the most active in this area, with far more publications and citations than any other country. The top institutions that have published the most works regarding this topic are Los Angeles, the University of Toronto in Canada, the Technical University of Dresden in Germany, etc. Bauer M. and Whybrow P.C. of the University of Los Angeles, and Frye Mark A. have higher publication volume and citation frequency, and these scholars have led research in this field. In terms of journals, the Journal of Affective Disorders, Bipolar Disorders, and the Journal of Clinical Psychiatry occupy the top 3 positions in terms of the number of articles issued, and Biological Psychiatry journals have a high citation rate. These findings suggest growing attention to this area and highlight its potential in the life sciences.

### 4.1. Countries/regions and their cooperation

According to the distribution of research countries, developed nations such as the United States, Germany, and the United Kingdom have a greater focus on research in this field. These countries have conducted earlier research in the field of thyroid hormone-related BD, and the United States occupies the first place with nearly 40% of the publications, indicating that its research in this field is more in-depth. There are the top 3 representative studies that have been cited with the highest frequency in the United States: a paper that demonstrates that miRNAs and their predicted effects are targets for the action of psychotherapeutic drugs by Zhou R^[11]^ (Citations: 252); another review linked thyroid hormones with the serotonergic system, suggesting that exogenous thyroid hormones may modulate affective disorders via serotonergic neurotransmission^[12]^ (Citations: 224); the third study reviewed previous research on neurobiological correlates and treatment response to depression in children, adolescents, and adults.^[13]^ China has also contributed to this research area with several representative studies. The representative literature is reported by Charles L. Bowden et al in 2010, which compared the efficacy and safety of sodium valproate and lithium in the treatment of BD patients with mania or mixed episodes^[14]^ (Citations: 44). He, B. et al studied the relationship between type II deiodinase gene polymorphisms and BD in a Chinese population from a pathophysiological perspective^[15]^ (Citations: 34), and Ja Y, 2015 studied the correlation of biochemical abnormalities in frontal white matter, hippocampus, and serum thyroid hormone levels in patients with the first episode of major depression from a neuropathological perspective^[16]^ (Citations: 26), while other countries, including Poland and Pakistan, have concentrated their publications in the last 5 years, making them emerging countries in the field of research. Two studies from Poland all showed that there may be a higher prevalence of thyroid dysfunctions in patients with bipolar and unipolar depression and that it is worth considering some kind of intervention regarding thyroid function in depressed individuals.^[17,18]^ Lithium-based medications are successfully used to treat BD. However, limitations in detecting lithium in organs and cells limit the ability to improve lithium-based treatments. IRFAN Ahmed et al from Pakistan developed laser-induced breakdown spectroscopy to detect lithium in biological tissues, which may potentially be adapted for use in the treatment of BD.^[19]^ Among the national cooperation networks, the United States, which has the highest number of publications, maintains close ties with Germany, China, the United Kingdom, and the Netherlands. Similarly, China also maintains close cooperative relationships with these countries.

### 4.2. Keywords

The keyword with the highest frequency of occurrence is “bipolar disorder,” followed by “thyroid function,” “thyroid hormone,” and “hypothyroidism.” The thyroid hormone is a hormone secreted by the thyroid gland, which acts on almost all cells in the body. The secretion of thyroid-stimulating hormone (TSH) is regulated by thyrotropin-releasing hormone (TRH), which is secreted by the hypothalamus. Stress, changes in environmental temperature, and certain diseases affect thyroid function through the release of TRH. On the other hand, tetraiodothyronine (T4) and triiodothyronine (T3) concentrations in the blood have a negative feedback effect on the release of both TSH and TRH.

In neurodevelopmental terms, researchers suggested in the 1990s that thyroid function may be set during fetal growth and infant feeding,^[20]^ and reduced plasma thyroxine levels coupled with an impaired response to thyrotropin-releasing hormone are associated with depression.^[21]^ A study by Thompson C in 2001 mentioned that fetal malnutrition predisposes men to depression in late adulthood and the mechanism may be mediated by the hypothalamic–pituitary–thyroid (HPT) axis.^[22]^ Disorders of the HPT axis are commonly reported in individuals with BD compared to the general population.^[23]^ Further studies have shown that HPT axis dysfunction is associated with neuropsychological and pathophysiological functions in BD, and that thyroid hormone abnormalities, cognitive dysfunction, and neurometabolic alterations in the prefrontal cortex–thalamic circuit occur simultaneously during the early course of BD type 2 depression.^[8,24]^ These studies provide a neurobiological perspective on the study of BD and thyroid hormones.

In the clinical work, we often find that patients with BD may present with changes in thyroid hormone, as Kupka RW argues that the effect of hypothyroidism was significant in studies using current, but not lifetime definitions of rapid cycling. Hypothyroidism may be associated with emotional instability in vulnerable patients.^[25]^ Studies have found a diminished response of TSH to TRH during mania^[26–28]^ without elevated serum thyroxine index^[29]^ and decreased serum T3 levels.^[30]^ Su Yousong et al found that thyroid function abnormalities in patients with remission and episode mania/hypomania primaries differed from those in patients with depression primaries, and that the episodic mania/hypomania primaries group had differences in T3, free triiodothyronine, and free thyroxine levels that were higher than in the depression priming group.^[31]^ Ozerdem A’s study shows a higher rate of TSH abnormality in patients with BD, particularly those taking lithium, compared to those with other psychiatric and medical conditions.^[32]^ In mood stabilizer maintenance therapy for BD, low levels of total serum thyroxine, FT4 and high levels of FT3 within the normal range are associated with the recurrence of depression. When mood stabilizers combined with antidepressants were used in the maintenance treatment of BD, high levels of FT3 within the normal range were related to the recurrence of mania.^[33]^ A higher FT4 level is associated with a reduced risk of BD, suggesting the importance of FT4 level in BD risk assessment and potential therapeutic target development.^[34]^ BD is closely related to thyroid dysfunction. Psychiatric medications have a greater or lesser effect on thyroid function, and thyroid hormone levels also influence the effect of medication. These findings suggest that thyroid function in BD patients differs significantly between depressive and manic episodes. In clinical practice, it is necessary to consider the differences in thyroid hormone levels in BD patients in different mood states for drug selection.^[10]^ However, some contrary studies, such as Li C, have shown that thyroid functions in BD patients did not significantly fluctuate between depressive and manic episodes.^[35]^ Possible factors contributing to the opposite result were fewer cases in the manic group.

Autoimmune is an important etiological hypothesis of BD, which has been verified both clinically (cytokines, immune cells, and other laboratory tests) and preclinically (brain slice neuroimmunostaining). There are also many studies on the effect of thyroid autoimmunity on BD, such as the study by Kupka, RW, which concluded that thyroid autoimmunity was highly prevalent in a sample of outpatients with BD, independent of lithium treatment. These variables appear to be independent risk factors for the development of hypothyroidism, especially in female patients with BD.^[36]^ His study also found that BD was associated with an acute phase response and cell-mediated activation of the immune system, and with an increased prevalence of antithyroid autoantibodies.^[37]^ Vonk R suggested that autoimmune thyroiditis is associated not only with BD itself but also with the genetic vulnerability in which the disorder occurs. Autoimmune thyroiditis marked by TPO-Abs is a possible endophenotype of BD.^[38]^ A study of offspring with BD found that they are more vulnerable to developing thyroid autoimmunity.^[39]^ Gan study suggests that TPO-Abs may be used as a biomarker for rapid cycling BD. Further exploring the underlying mechanism might help understand the nature of rapid cycling BD and find new therapeutic targets.^[40]^ Additional studies suggest that autoimmune processes precede the onset of schizophrenia, as well as nonaffective psychosis and BD.^[41]^

### 4.3. Clinical applications and mechanisms of thyroxine in BD

At present, the treatment of BD has gradually expanded to include the use of thyroid hormones. In most clinical guidelines, lithium remains the first-line prophylactic therapy for BD type 1; however, studies have shown that patients with BD type 1 undergoing long-term lithium treatment often exhibit a higher incidence of thyroid dysfunction.^[42–45]^ Given these frequent thyroid abnormalities associated with lithium therapy, increasing attention has been directed toward the potential therapeutic role of thyroid hormones in BD. The findings provide evidence that the administration of supraphysiologic thyroid hormone improves depressive symptoms in patients with BD by modulating the function of the anterior limbic network.^[46]^ Bauer M concluded that bipolar depressed patients have abnormal function in prefrontal and limbic areas. L-T4 may improve mood by affecting circuits involving these areas, which have previously been implicated in affective disorders.^[47]^ Kelly T study concluded that high-dose thyroid hormones would be a first treatment that decreases the considerable medical morbidity and mortality associated with BD.^[48]^ Results of a study indicated no increased risk for cardiovascular disorders during treatment with supraphysiologic doses of L-T4 in patients with refractory mood disorders.^[49]^ Many clues suggest that T3 could augment and accelerate the therapeutic response not only to antidepressants but also to lithium and perhaps to other treatment regimens, which could prevent rapid cycling of BD as well as prevent relapse during the first years of treatment.^[50]^ Pilhatsch M study failed to find differences in anxiety changes in patients with bipolar depression treated with supraphysiologic doses of L-T4 or placebo. Comorbid anxiety symptoms should not be considered a limitation to the treatment of patients with supraphysiologic doses of L-T4-refractory bipolar depression.^[51]^ Changes in normal range TSH and FT4 levels did not affect the risk of major depressive disorder and its subtypes, nor did they affect minor depressive symptoms. This suggests that depressive symptoms should not be attributed to small changes in thyroid function. The borderline association with the risk of BD and BD type 1 suggests that further clinical studies should investigate the effect of thyroid hormone therapy on BD.^[52]^

## 5. Conclusion

This study provides a comprehensive bibliometric analysis of research on thyroid hormones and BD, revealing a steady increase in publications from 1998 to 2022. The United States and China were the leading contributors in this field, with major institutions such as the University of California, Los Angeles, and the University of Toronto playing significant roles. The analysis of research hotspots highlighted a shift in focus from pathophysiological mechanisms and mood disorder classification to pharmacological mechanisms, hormonal treatments, and the optimization of clinical interventions. Notably, there has been growing interest in thyroid hormone augmentation therapy, particularly supraphysiologic doses of L-T4 for treatment-resistant mood disorders. Keyword burst analysis identified key terms such as “risk,” “affective illness,” and “bipolar affective disorder,” with the strongest burst intensity observed for “risk” in 2013, emphasizing the increasing focus on genetic predisposition, treatment-induced thyroid dysfunction, and comorbidity risks. This evolving research trend underscores the interdisciplinary nature of BD studies, linking psychiatry, endocrinology, and neurobiology, and highlights the need for personalized treatment approaches that integrate thyroid function monitoring for improved management of BD.

In a word, our bibliometric study identifies some key research hotspots in thyroid hormone and BD, which may provide a structured overview of how BD research has evolved and may help guide future studies aimed at improving quantitative assessment, mechanistic understanding, and clinical application.

## 6. Limitations

Our study also has some limitations. Firstly, the scope of our analysis was confined to publications indexed within the WoSCC, potentially omitting relevant articles in nonEnglish publications and regional journals or databases (such as Scopus, PubMed, or China National Knowledge Infrastructure), limiting the completeness of literature coverage. Secondly, due to the limited number of publications, future research on keyword emergence analysis may be warranted. Thirdly, further exploration of whether the framework highlights under-explored pathways or key research priorities from the current bibliometric analysis needs to be explored. Finally, the manual removal of papers unrelated to the study by the investigator may lead to selection bias.

## Author contributions

**Conceptualization:** Ligang Wang.

**Formal analysis:** Shenghai Wang.

**Investigation:** Xiuhua Song, Lei Yi, Shenghai Wang.

**Supervision:** Shenghai Wang, Ligang Wang.

**Validation:** Xiuhua Song, Lei Yi.

**Visualization:** Xiuhua Song, Lei Yi.

**Writing – original draft:** Xiuhua Song.

**Writing – review & editing:** Ligang Wang.
